# β‐Glucan attenuates cognitive impairment of APP/PS1 mice via regulating intestinal flora and its metabolites

**DOI:** 10.1111/cns.14132

**Published:** 2023-03-08

**Authors:** Qiwei Zhang, Wei Zhao, Yue Hou, Xinxin Song, Haiyang Yu, Jinghe Tan, Yanmeng Zhou, Han‐Ting Zhang

**Affiliations:** ^1^ College of Animal Science and Veterinary Medicine Shandong Agricultural University Tai‐an City China; ^2^ Institute of Pharmacology Shandong First Medical University and Shandong Academy of Medical Sciences Taian China; ^3^ Department of Pharmacology, School of Pharmacy Qingdao University Qingdao China

**Keywords:** AD, brain gut axis, intestinal flora metabolites, neuroinflammation, β‐Glucan

## Abstract

**Background:**

The intestinal flora has been shown to be involved in the progression of Alzheimer's disease (AD) and can be improved by β‐glucan, a polysaccharide derived from Saccharomyces cerevisiae, which affects cognitive function through the intestinal flora. However, it is not known if this effect of β‐glucan is involved in AD.

**Method:**

This study used behavioral testing to measure cognitive function. After that, high‐throughput 16 S rRNA gene sequencing and GC–MS were used to analyze the intestinal microbiota and metabolite SCFAs of AD model mice, and further explore the relationship between intestinal flora and neuroinflammation. Finally, the expressions of inflammatory factors in the mouse brain were detected by Western blot and Elisa methods.

**Results:**

We found that appropriate supplementation of β‐glucan during the progression of AD can improve cognitive impairment and reduce A β plaque deposition. In addition, supplementation of β‐glucan can also promote changes in the composition of the intestinal flora, thereby changing the flora metabolites in the intestinal content and reduce the activation of inflammatory factors and microglia in the cerebral cortex and hippocampus through the brain‐gut axis. While reducing the expression of inflammatory factors in the hippocampus and cerebral cortex, thereby controlling neuroinflammation.

**Conclusion:**

The imbalance of the gut microbiota and metabolites plays a role in the progression of AD; β‐glucan blocks the development of AD by improving the gut microbiota and its metabolites and reducing neuroinflammation. β‐Glucan is a potential strategy for the treatment of AD by reshaping the gut microbiota and improving its metabolites.

## Graphical abstract

Gut microbiota is involved in the regulation of Alzheimer's disease(AD) related neuroinflammation through the brain‐gut axis. Yeast‐derived β‐glucan may prevent AD development through effects on gut microbiota and its metabolites.

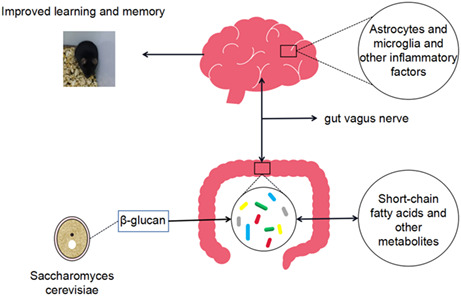

## 1 INTRODUCTION

Alzheimer's disease (AD) is a progressive neurodegenerative disease characterized by memory and cognitive dysfunction. The neuropathological features of AD include amyloid‐β (Aβ) plaques and hyperphosphorylated tau protein tangles.^1^ Although people have made great efforts to treat AD and reveal its mechanism in the past decades, there is no effective treatment method in clinical practice.^2^, ^3^ Recently, studies on the brain gut axis have found that there is a dynamic interaction between the brain and intestinal microbiota.^4^, ^5^ Brain gut axis may play an important role in brain functions (such as cognitive learning and memory) through vagus nerve, hormone signal and metabolism of specific molecules.^6^, ^7^ Studies from AD mouse models^8^ and AD patients^9^ have demonstrated that the disturbance of intestinal microbial diversity produces neuroinflammation and amyloidosis.^10^, ^11^ In addition, the pathology of AD mouse models can be restored by transferring healthy microbiota^12^ and, similarly, the cognitive dysfunction of AD patients can be improved by restoring the gut microbiota.^13^ This indicates that treatment of specific intestinal microbiota of AD patients may alleviate the neurological symptoms of AD, and brain gut axis may be a new direction to prevent or delay the progress of AD.

It has been demonstrated in studies from animals and humans that the intestinal microbiota is involved in the activation of AD‐related microglia and the regulation of neuroinflammation.^14^, ^15^ In addition, it is necessary to understand the relationship between specific metabolites of the intestinal flora and neuroinflammation during the progression of AD. The present study aimed to explore the mechanism link between the intestinal environment and the progression of AD. We used yeast β‐glucan, a polysaccharide derived from the yeast of Saccharomyces cerevisiae, to interfere with the intestinal flora. As an economic by‐product of yeast, β‐glucan is high‐yield and easy‐to‐obtain. It has been processed into functional food and used for preventive applications.^16^ It is worth noting that different sources and different molecular weights of β‐glucan have different biological activities.^17^, ^18^ Wang et al. showed that circulating total cholesterol in adults with mild hypercholesterolemia could be improved by high molecular weight β‐glucan rather than low molecular weight β‐glucan.^19^ Another study found that the antioxidant activity of β‐glucan in barley increased with increasing molecular size.^20^ These results indicate that the bioactivity of β‐glucan is closely related to its molecular size.

Research on β‐glucan is mainly focused on the regulation of immunity, insulin and anti‐tumor activity,^21^ while recently, it has been demonstrated that polysaccharides improve the composition of the intestinal flora and affect the production and aggregation of Aβ.^22^ In addition, recent studies have reported that β‐glucan treatment exerts neuroprotective and anti‐inflammatory effects by increasing metabolites such as short‐chain fatty acids (SCFA) in mice and humans,^23^, ^24^ but the role of β‐glucan in AD and the relevant mechanisms remain largely unknown. Therefore, it was necessary to characterize the role of β‐glucan in the mediation of AD and identify the underlying mechanisms of the complex relationship between yeast β‐glucan, intestinal flora, neuroinflammation, and cognitive function.

In the present study, we investigated whether different molecular weight yeast β‐glucan alleviated the cognitive dysfunction of APP/PS1 mice by restoring intestinal function. In addition, we examined the dynamic changes in intestinal flora and the relationship between cognitive function and neuroinflammation. The relationship between intestinal flora and AD was also determined.

## 2 MATERIALS AND METHODS

### 2.1 Animals

APP/PS1 mice can express a fusion of mutant human senescentin (DeltaE9) and mouse amyloid preprotein (APPswe); the expression of these two genes is initiated by the mouse prion protein promoter.^25^ This mutation can cause early‐onset of AD and has become an animal model of AD based on this cause. This genetically superior mouse line has obvious changes in the composition of the gut flora, which makes it an ideal model for studying the association between AD and the brain‐gut axis.^26^ Therefore, adult male C57BL/6J and APP/PS1 mice (Shandong Skobas Biotechnology) at 6 months of age, weighing 20–25 g, were used in the experiments. Animals were housed in plastic cages with controlled temperature (24 ± 2°C) and humidity (40%–50%) on a 12‐h light/dark cycle. Mice had free access to food and water. The experimental protocol was approved by the Committee of Animal Experimental Ethics of Shandong First Medical University.

### 2.2 Experimental design

First of all, mouse colon tissue cultures were prepared for assays. Mice were randomly divided into four groups (*n* = 8 per group): C57BL/6J mice (WT), APP/PS1 mice, APP/PS1 mice treated with small‐molecular β‐glucan (S‐β‐Glu) or macro‐molecular β‐glucan (M‐S‐Glu) each at the dose of 100 mg/kg by oral gavage once per day for 1 month. The other two groups took the same volume of saline orally for 1 month. After the completion of treatment, all mice were sacrificed, the colon tissues (0.1 g) were inoculated into an anaerobic meat liver soup medium and cultured at 37°C for 24 h. The cultures were then stored at −80°C for the subsequent experiments.

Next, we tested whether the cultivation of the mouse own intestinal flora would affect the cognitive ability of the same animal. Mice were randomly divided into four groups (*n* = 8 per group): WT or APP/PS1 + culture, WT or APP/PS1+ vehicle, i.e., WT or APP/PS1 mice received their own intestinal content cultures at 0.3 mL/kg or the same dose of vehicle (anaerobic meat liver soup medium) by rectal administration for 2 months.

Finally, we divided the experiment into six groups with 8 mice each: WT, APP/PS1, S‐β‐Glu‐treated mice with intestinal flora culture (S‐Glu‐I)、M‐β‐Glu‐treated mice with intestinal flora culture (M‐Glu‐I)、high‐dose S‐β‐Glu‐treated mice with intestinal flora culture (HS‐Glu‐I), and high‐dose M‐β‐Glu‐treated mice with intestinal flora culture (HM‐Glu‐I). The S‐Glu‐I and M‐Glu‐I groups were given with 0.3 mL/kg of the corresponding colon cultures through the rectum, while the HS‐Glu‐I and HM‐Glu‐I groups were given with 0.6 mL/kg. WT and APP/PS1 mice administered its own intestinal cultures at 0.3 mL/kg. All mice were sacrificed 10 d after behavioral testing.

### 2.3 Morris water maze (MWM) test

The cognitive function of mice was evaluated using the MWM as previously described.^27^ Mice were trained for 5 consecutive days to position the hidden platform in one of the four quadrants of the water in the circular tank. In order to exclude variations caused by the circadian rhythm, animals were trained and tested each day between 10:00 AM and 5:00 PM. During each trial, the mouse was first allowed to position itself on the platform for 60 s. If it failed to find the platform within 60 s, mice were placed on the platform for 10 s and the time required to reach the platform (escape latency) was recorded as 60 s. On day 6, the experimenter removed the platform and recorded the time spent in the target quadrant and the number of crossings into the platform within 60 s. The tracking information was processed by the Topscan Package (Clever Sys Inc.).

### 2.4 Step‐through passive avoidance test

The test is designed with the habit of repelling darkness and avoiding light by rodents^28^ and was conducted following our previous procedures with minor modifications.^29^ The device was composed of two identical boxes (28 × 15.5 × 16 cm):one was dark and the other was bright. There was a small hole connecting the two boxes with a valve control switch; the floor was copper grid on the dark side which could deliver foot shocks. The experiment lasted for 3 d. On the first day, the mice were placed in a box to adapt to the environment without any stimulus interference. After 24 h for learning and training, the mice were placed in the dark box. When the limbs and tail were completely put into the dark box, the door was immediately closed and an electric shock (0.25 mA, 3 s was introduced, followed 10 s later by another electric shock (0.25 mA, 2 s). Memory was tested on the third day using the same procedure as that on the first day.

### 2.5 Object recognition test

This was performed as previously described.^30^ Each mouse was allowed to freely move in an empty box for 5 min as habituation while spontaneous activities were recorded at the same time. Twenty‐four hours later, the mouse was placed alone in the center of the box. There were two identical objects (Lego bricks) at the two opposite corners of the box. The cumulative time spent exploring each object was recorded for 5 min. Exploration is defined as actively touching or facing (within 2 cm) an object. The memory ability was tested 24 h after training using the same procedure, except that one of the familiar objects was replaced with a completely new one. The exploration time of each object [Tf and Tn for familiar (f) and new (n) objects, respectively] was used to determine the recognition index (RI): RI = Tn/(Tn + Tf).

### 2.6 Y‐maze test

This was performed following the processes published earlier with some modifications.^31^ The Y‐maze consists of three identical arms (A, B, C) with different hints. The mouse was placed in the starting central area connecting the three arms and the sequence of the probe arms (for example, ABCBA) was recorded using a video camera, which was used to record the total number of arm entries and alternation behaviors. The accuracy of the Y maze performance was the ratio between correct alternation and total alternations.

### 2.7 ELISA assay

Blood samples were collected from the orbital sinus and proteins were isolated from brain tissues. The concentrations of IL‐1β and IL‐6 in the hippocampus, cortex, and serum were determined using mouse IL‐1β (ml063132) and IL‐6 (ml063159) ELISA kits, respectively (Shanghai Enzyme‐linked Biotechnology, Shanghai, China), according to the manufacturer's instructions. Absorbance at 450 nm was recorded with a multifunctional microplate reader (TECAN, Switzerland).

### 2.8 Western blotting

The hippocampal tissues were homogenized in ice‐cold lysis buffer, and then the homogenate was denatured with the buffer at 100°C for 10 min. The protein concentration in the extract was determined by the BCA protein detection kit (Beyotime, China). The prepared protein was separated by 8%–12% sodium dodecyl sulfate‐polyacrylamide gel (SDS/PAGE) and transferred to nitrocellulose (NC) filter membrane. After incubating with blocking solution (5% skimmed milk powder diluted with 0.1% Tween‐20 in Tris buffered saline) at room temperature for 30 min, the NC membrane was incubated with the primary antibodies at 4°C overnight. The membrane was then washed three times with TBST for 10 min and incubated with the appropriate secondary antibodies (mouse/rabbit) for 1 h at room temperature. The density of each signal was measured by the Image J software.

### 2.9 Immunofluorescence analysis

The whole brain was collected, fixed in 4% paraformaldehyde buffer at 4°C overnight, dehydrated with graded ethanol, and embedded in paraffin, before cutting into slices of 4 μm in thickness, which were mounted on glass slides. The slices were dried at 60°C in an oven and stored at room temperature. The sections were deparaffinized, rehydrated, and subjected to antigen retrieval, followed by rinsing in distilled water. The sections were blocked in 3% bovine serum albumin (BSA; G5001, Servicebio) for 30 min, and then incubated with IBA‐1 (ab178846, 1:300, Abcam) and GFAP antibodies (ab7260, 1:300, Abcam) overnight at 4°C. The next morning, the sections were washed with distilled water, and incubated with horseradish peroxidase‐conjugated goat anti‐rabbit secondary antibodies (GB23303, 1:200, Servicebio) in the dark at room temperature for 1 h, followed by 40,6‐diamididine and 2‐phenylindole (DAPI, G1012, Servicebio) together for 10 min. The sections were then washed in phosphate buffered saline (PBS), incubated with autofluorescence quenching reagent (GB1221, Servicebio) for 5 min, and washed again in PBS, before mounting on a microscope slide with anti‐fading mounting medium. The slices were observed under a fluorescence microscope (Axioscope 5, Carl Zeiss), images were capture with a slice scanner (Panoramic MIDI, Danjier) using Image‐Pro Plus software version 6.0 (Media Cybernetics Processing Company).

### 2.10 Immunochemistry

The slices were dried at 60°C in the oven and stored at room temperature. The sections were deparaffinized, rehydrated, and subjected to antigen retrieval, and then rinsed in distilled water. The sections were blocked with 3% bovine serum albumin (BSA; G5001, Servicebio) for 30 min, and then incubated with anti‐Aβ1‐42 antibody (Ag22408, 1:200, Proteintech) at 4°C overnight. The next morning, the secondary antibodies (HRP labeled) of the corresponding species were added to cover the tissues, and incubated at room temperature for 50 min. The slides were then placed in PBS (PH7.4) for decolorization. After the sections were dried a little, freshly prepared DAB chromogenic solution was dripped in the circle. The color development time was controlled under the microscope. The positive color was brown. Rinse the sections with double distilled water to stop the color display. Hematoxylin was counter‐stained for about 3 min, washed with double distilled water, differentiated with hematoxylin differentiation solution for a few seconds, rinsed again with double distilled water, and as the hematoxylin blue solution turned blue, rinsed with running water. Finally, the slices were placed in the following conditions for dehydration and transparent: 75% alcohol for 5 min ‐ 85% alcohol for 5 min ‐ anhydrous ethanol I for 5 min ‐ anhydrous ethanol II for 5 min ‐ n‐butanol for 5 min ‐ xylene I for 5 min. Sections were removed from toluene and allowed to dry before the slides were covered with neutral gum.

### 2.11 DNA extraction and sequencing of colon content samples

All stool samples were stored at −80°C before DNA extraction and analysis. The detection method of intestinal flora 16 s according to the previous description.^32^, ^33^ The sequence was amplified using the corresponding primers, and the PCR products were confirmed by 2% agarose gel electrophoresis. In the entire DNA extraction process, ultrapure water was used instead of sample solution to eliminate the possibility of false positive PCR results. The PCR products were purified by AMPure XT beads (Beckman Coulter Genomics) before Qubit (Invitrogen) quantified. The amplicon pool was used for sequencing, and the size and quantity of the amplicon library were evaluated using the Agilent 2100 Bioanalyzer (Agilent) and Illumina (Kapa Biosciences) library quantification kits. The library was sorted using the NovaSeq PE250 platform.

### 2.12 Extraction and analysis of SCFAs and bile acids in colon contents

Metabolomics assay method referenced from previous studies.^34^, ^35^ A standard curve was established for short‐chain fatty acids and bile acids using the corresponding standards. After accurately weighing the stool samples, 500 μL of methanol–water solution (containing 0.1% HCl, 20% H_2_O) was added. Then the isotopic internal standard acetic acid‐d4 was added and pulverized with a freezer pulverizer, followed by sonication in an ice bath for 10 min. The samples were vortexed and the supernatant (200 μL) was taken for GC–MS/MS analysis after centrifugation at 12,000 rpm, 4°C for 5 min.

### 2.13 Statistical analyses

Statistical analysis was carried out by using GraphPad Prism 6.0 (GraphPad software). All data are expressed as mean ± standard error of the mean (S.E.M). The Shapiro–Wilk test was used for the normality of data, and the Bartlett test was used for the homogeneity of the variance. The difference between two normal data were analyzed by using two‐tailed *t*‐test, and the non‐normal data were analyzed by using rank sum test. The differences among more than two groups were analyzed using one‐way analysis of variance (ANOVA) followed by Dunnett's test for each two groups. Significance was set at *p* < 0.05 for all tests.

## 3 RESULTS

### 3.1 β‐Glucan attenuated cognitive impairment in APP/PS1 mice

**FIGURE 1:**
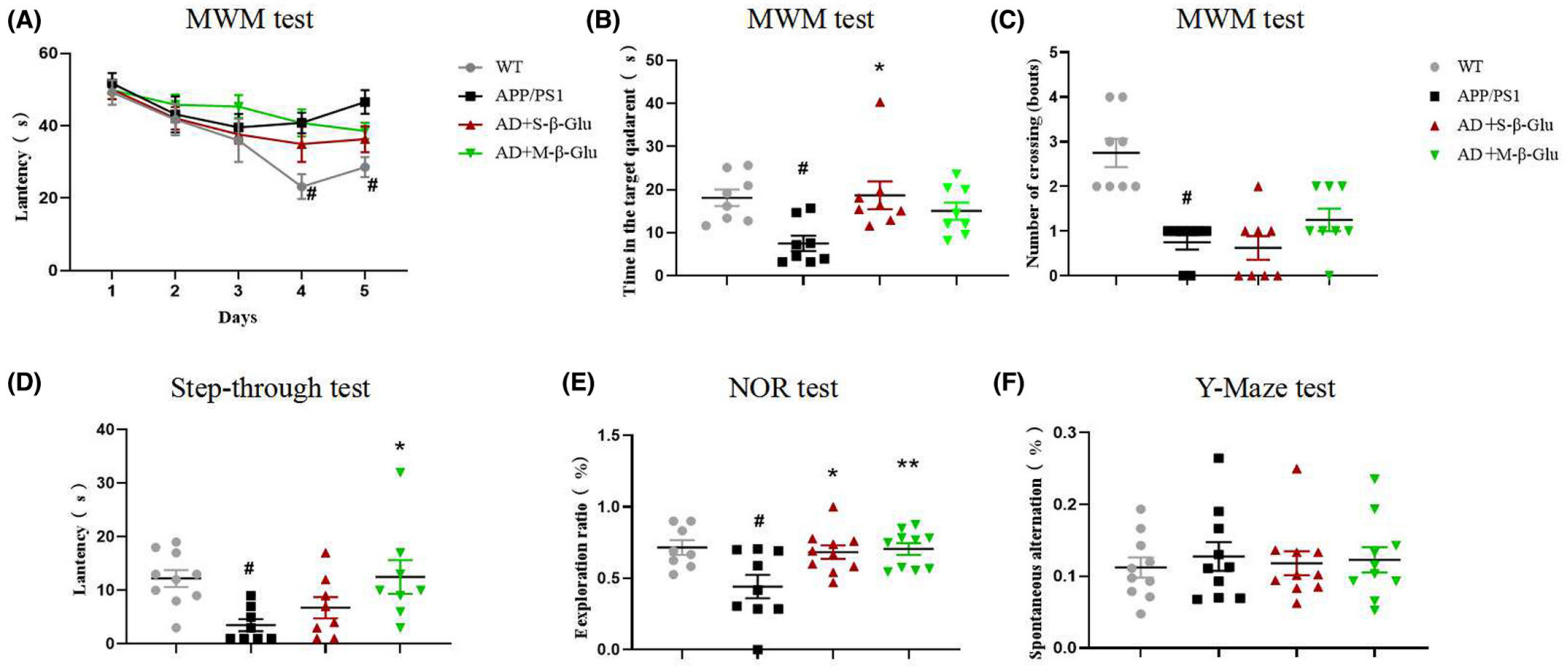
β‐Glucan treatment attenuated impairment of learning and memory in APP/PS1 mice using the Morris Water maze (MWM) (A‐C), step‐through passive avoidance (D), and novel object recognition (NOR) (E) tests. (A) The effect of β‐glucan on the average escape latency (s) to the hidden platform during the 5‐d acquisition training. (B) The effect of β‐glucan on the time spent in the target quadrant during the probe trial. (C) The effect of β‐glucan on the number of crossings into the platform during the probe trial in the MWM. (D) The effect of β‐glucan on the average latency (s) to the dark compartment in the step‐through passive avoidance test. (E) The effect of β‐glucan on the discrimination index in NOR. Small‐ or macro‐molecular β‐glucan (S‐β‐Glu or M‐S‐Glu, respectively) was given at a dose of 100 mg/kg by oral gavage once per day for 1 month. Data shown are means ± SEM. *p < 0.05, **p < 0.01 vs. APP/PS1; #*p* < 0.05 vs. WT; *n* = 8–10 mice/group.

Compared with WT, APP/PS1 (AD) mice had significant impairment of memory and cognition, as indicated by the longer time required to reach the escape platform during the acquisition training (Figure 1A), the decreased number of crossings in the target quadrant (Figure 1B,C) in the MWM test, the shortened latency to enter the dark compartment in the step‐through test (Figure 1D), and decreased novel object exploration in the object recognition test (Figure 1E). Treatment with S‐β‐Glu or M‐β‐Glu reversed the impairment of learning and memory in APP/PS1 mice in all the behavioral measurements except for the crossings in the MWM test (Figure 1A–E). Unexpectedly, the Y‐maze test did not show any response to the AD mice, regardless of the treatment (Figure 1F).

### 3.2 β‐Glucan delayed the development of Aβ plaque deposition and neuroinflammation

**FIGURE 2:**
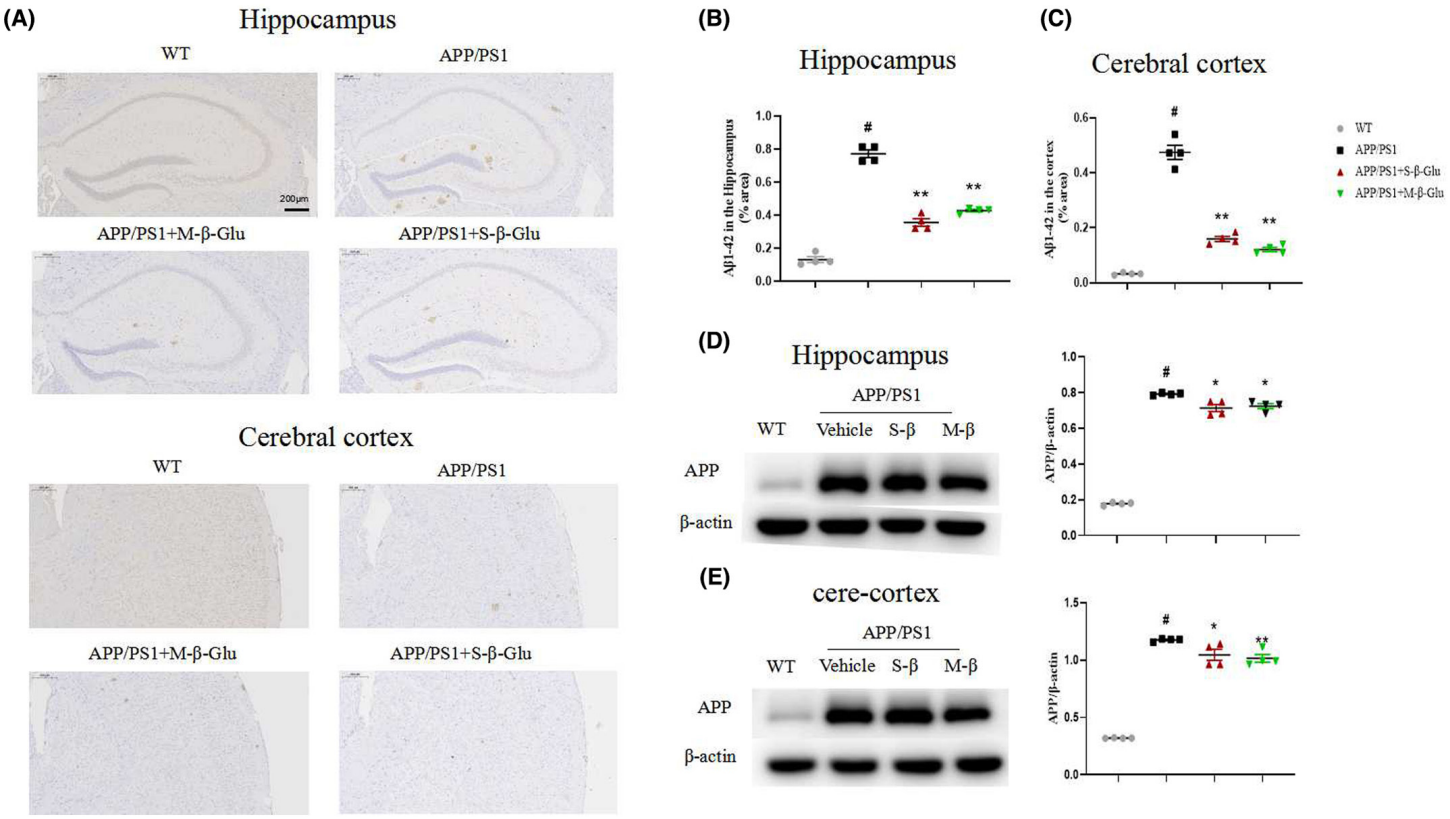
β‐Glucan treatment alleviated AD‐related pathological changes in the hippocampus and cerebral cortex of APP/PS1 mice using H&E staining (A–C) and Western blotting (D,E). (A) H&E staining of the hippocampus and cerebral cortex. Scale bar represents 200 μm. (B,C) Quantification of Aβ1–42 by H&E staining in the hippocampus (B) and cerebral cortex (C). (D,E) Western blotting imaging (left) and quantification (right) of APP proteins in the hippocampus (D) and cerebral cortex (E). S‐β‐Glu or M‐S‐Glu was given by oral gavage at 100 mg/kg once per day for 1 month. Bars shown are means ± SEM. *p < 0.05, **p < 0.01 vs. APP/PS1; #*p* < 0.05 vs. WT; *n* = 4 mice/group.

Immunohistochemical detection of Aβ plaque deposition in the brain revealed that the two types of β‐glucan significantly reduced Aβ plaque deposition in the hippocampus and cerebral cortex of APP/PS1 mice (Figure 2A–C). Consistent with this, Western blotting showed that β‐glucan also reduced the expression of APP in the hippocampus and cerebral cortex of APP/PS1 mice (Figure 2D,E).

**FIGURE 3:**
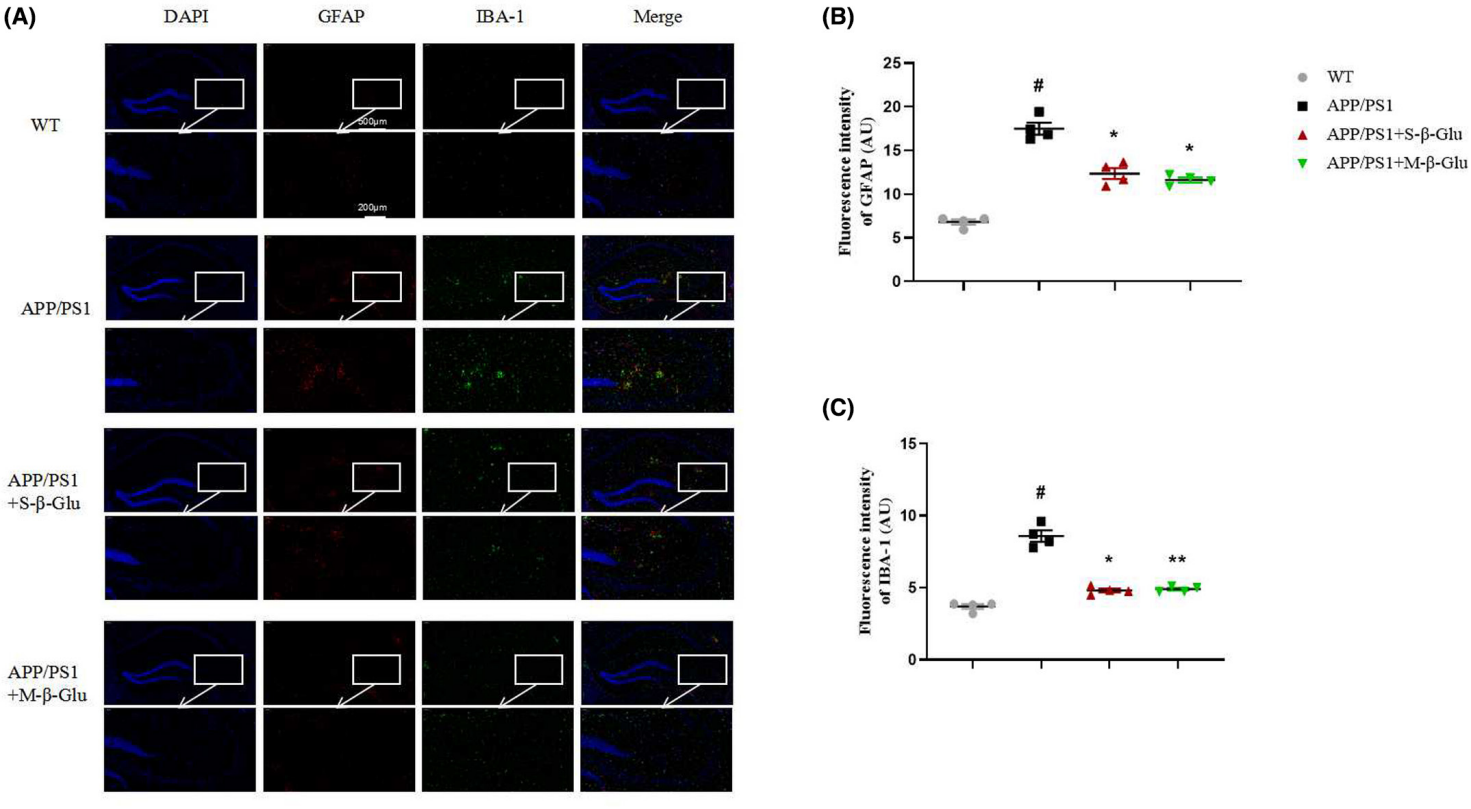
β‐Glucan treatment attenuated astrocyte and microglia activation of APP/PS1 mice using immunofluorescence (A–C). (A) Immunostaining of hippocampal slices with anti‐GAFP (red) and anti‐IBA1 (green). Nucleus staining was conducted with DAPI (blue). (B) The fluorescence intensity of GFAP, a marker of astrocyte activation. (C) The fluorescence intensity of IBA‐1, a marker of microglia activation. S‐β‐Glu or M‐S‐Glu was given by oral gavage at 100 mg/kg once per day for 1 month. Scale bars represent 500 μm (upper) or 200 μm (lower in magnification). Bars shown are means ± SEM. *p < 0.05, **p < 0.01 vs. APP/PS1; #*p* < 0.05 vs. WT; *n* = 4 mice/group.

**FIGURE 4:**
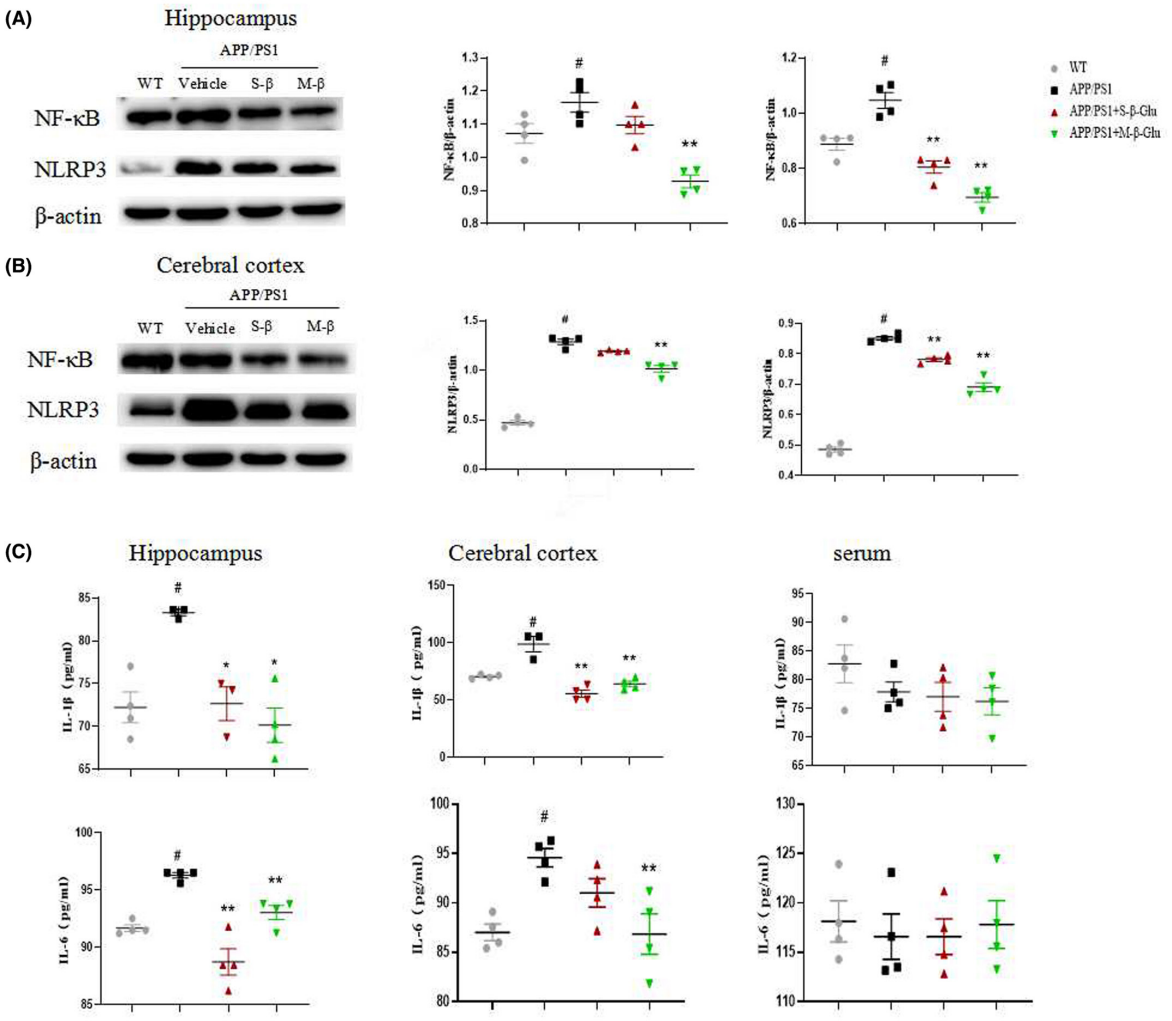
β‐Glucan treatment attenuated inflammatory responses in APP/PS1 mice using Western blotting (A–D) and ELISA (E–G). (A, B) Western blotting imaging (A) and quantification (B) of NF‐κB (left) and inflammasome NLRP3 (right) in the hippocampus. (C, D) Western blotting imaging (C) and quantification (D) of NF‐κB (left) and NLRP3 (right) in the cerebral cortex. (E, F) The effect of β‐glucan on the content of IL‐1β (upper) and IL‐6 (lower) in the hippocampus (E) and cerebral cortex (F). (G) The effect of β‐glucan on the content of IL‐1β (upper) and IL‐6 (lower) in the serum. Bars shown are means ± SEM. *p < 0.05, **p < 0.01 vs. APP/PS1; #*p* < 0.05 vs. WT; *n* = 4 mice/group.

We determined the activation of microglia and astrocytes in the mouse brain using immunofluorescence. Compared with the WT, APP/PS1 mice showed obvious inflammatory responses, as demonstrated by increased GFAP‐ and IBA‐1‐labeled cells (Figure 3A–C) and expression of NF‐kB and NLRP3 in the hippocampus and cerebral cortex of APP/PS1 mice (Figure 4A–D), indicating activation of astrocytes and microglia and inflammatory responses. Consistent with these, IL‐1β and IL‐6 were also increased in the hippocampus and cerebral cortex in APP/PS1 mice (Figure 4E,F). All these changes were attenuated by S‐β‐Glu and/or M‐β‐Glu. By contrast, there was no change in serum IL‐1β and IL‐6 in the four groups of mice (Figure 4G).

### 3.3 β‐Glucan restored intestinal flora imbalance in APP/PS1 mice

**FIGURE 5:**
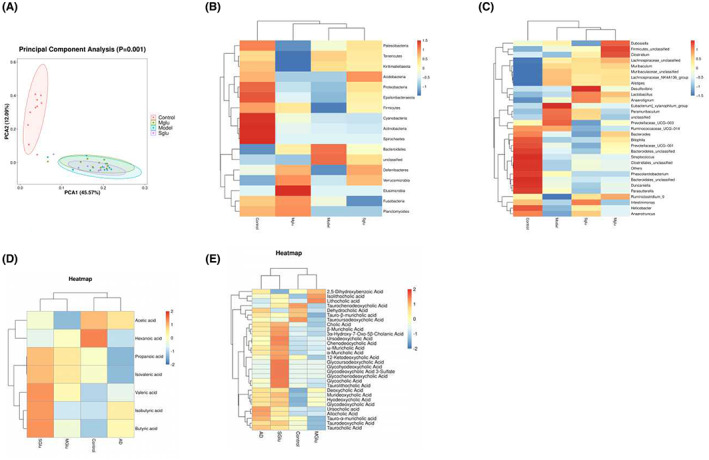
Changes in the intestinal flora and its metabolites in wild type (WT) and APP/PS1 mice treated with or without S‐β‐glucan or M‐β‐glucan. (A) Principal Component Analysis (PCA) of the similarities and differences between groups of biological replicates. (B,C) Heatmap of the differences in microbial species between groups at the phylum (B) and species (C) levels. (D,E) Heatmap of the differences in the contents of short‐chain fatty acids (SCFAs) (D) and bile acid (E) between groups. The color intensity represents the quantity (blue for negative and red for positive).

In order to evaluate the changes in the intestinal flora of APP/PS1 mice and the influence of yeast β‐glucan intake on the intestinal flora, the intestinal bacteria of the mouse colon contents were analyzed using 16 S rRNA gene sequencing. Principal component analysis (PCA), which preliminarily reflects the dispersion and aggregation distribution of samples after different treatments, was used to determine the similarity of the microbial communities in the four groups of samples (Figure 5A). The results showed that APP/PS1 mice had obvious changes in intestinal flora, with significantly reduced types of intestinal flora. At the phylum level, APP/PS1 mice displayed significant decreases in the number of intestinal flora relative to WT controls, which was attenuated after treatment with S‐β‐Glu or M‐β‐Glu (Figure 5B). Compared with wt mice, APP/PS1 mice showed a significant increase in bacteroidetes and a significant decrease in Firmicutes, Proteobacteria and actinomycetes. At the same time, there was a degree of reversal after glucan supplementation. At the species level, APP/PS1 mice had reduced bacterial species diversity, which was recovered after supplementation with β‐glucan (Figure 5C).

We then used targeted metabolomics technology to detect SCFA and bile acids in the colon of mice. It was found that there was a significant difference in SCFA between APP/PS1 and WT mice, that is, most of the SCFA contents in APP/PS1 mice were reduced. Supplementing S‐β‐Glu increased the content of propionic acid, butyric acid, and valeric acid in the colon of APP/PS1 mice, while M‐β‐Glu increased the content of propionic acid and valeric acid (Figure 5D). Compared with WT, APP/PS1 mice showed increases in the content of secondary bile acids in the colon, which was attenuated by supplementation with β‐glucan (Figure 5E).

### 3.4 Intestinal flora cultures of β‐glucan‐treated mice showed improvement in cognitive impairment of APP/PS1 mice

**FIGURE 6:**
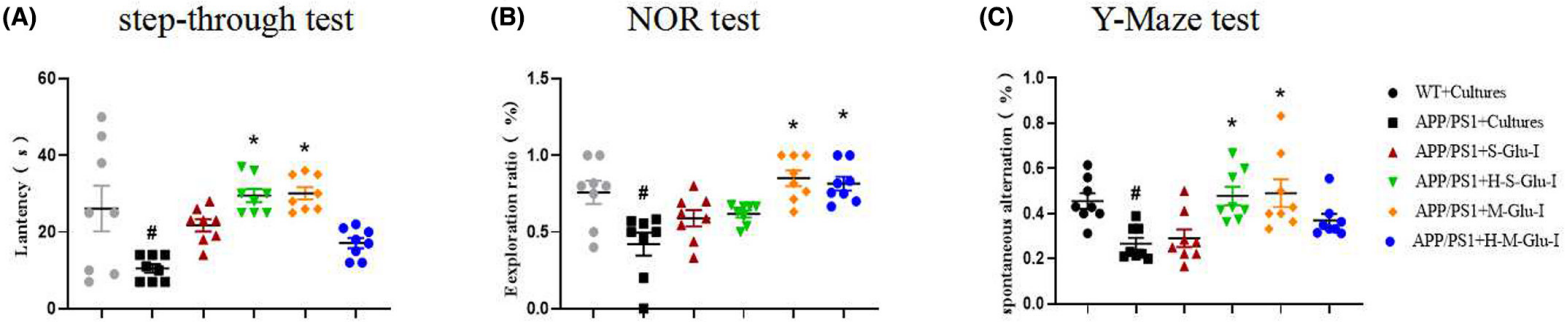
Gut microbiota cultures of β‐glucan‐treated mice attenuated impairment of learning and memory in APP/PS1 mice using the step‐through passive avoidance, novel object recognition (NOR), and Y‐maze tests. (A) The effect of intestinal flora cultures on the average latency (s) to the dark compartment in the step‐through passive avoidance test. (B) The effect of intestinal flora cultures on the discrimination index in the NOR test. (C) The effect of intestinal flora cultures on the spontaneous alteration in the Y‐maze test. Intestinal flora cultures were given at a dose of 0.3 mL/kg or 0.6 mL/kg by rectal administration once per day for 2 months. Data shown are means ± SEM. *p < 0.05 vs. APP/PS1; #*p* < 0.05 vs. WT; *n* = 8 mice/group.

To determine the effect of intestinal flora cultures on cognition of APP/PS1 mice, we treated APP/PS1 mice with intestinal flora cultures from mice administered β‐glucan. In the behavioral tests including the step‐through passive avoidance (Figure 6A), NOR (Figure 6B), and Y‐maze (Figure 6C), APP/PS1 mice showed significant impairment of memory, which was consistent with the previous result (Figure 1). Significant improvement of learning and memory was observed in mice after supplementation with intestinal flora cultures (Figure 6). To exclude the potential influence of the mouse's own intestinal flora culture, we administered its own intestinal flora cultures or vehicle to WT and APP/PS1 mice for comparison. WT and APP/PS1 mice did not show any significant changes in behavior in the memory tests (Figure S1).

### 3.5 Intestinal flora cultures of β‐glucan‐treated mice delayed the development of Aβ plaque deposition and neuroinflammation in APP/PS1 mice

**FIGURE 7:**
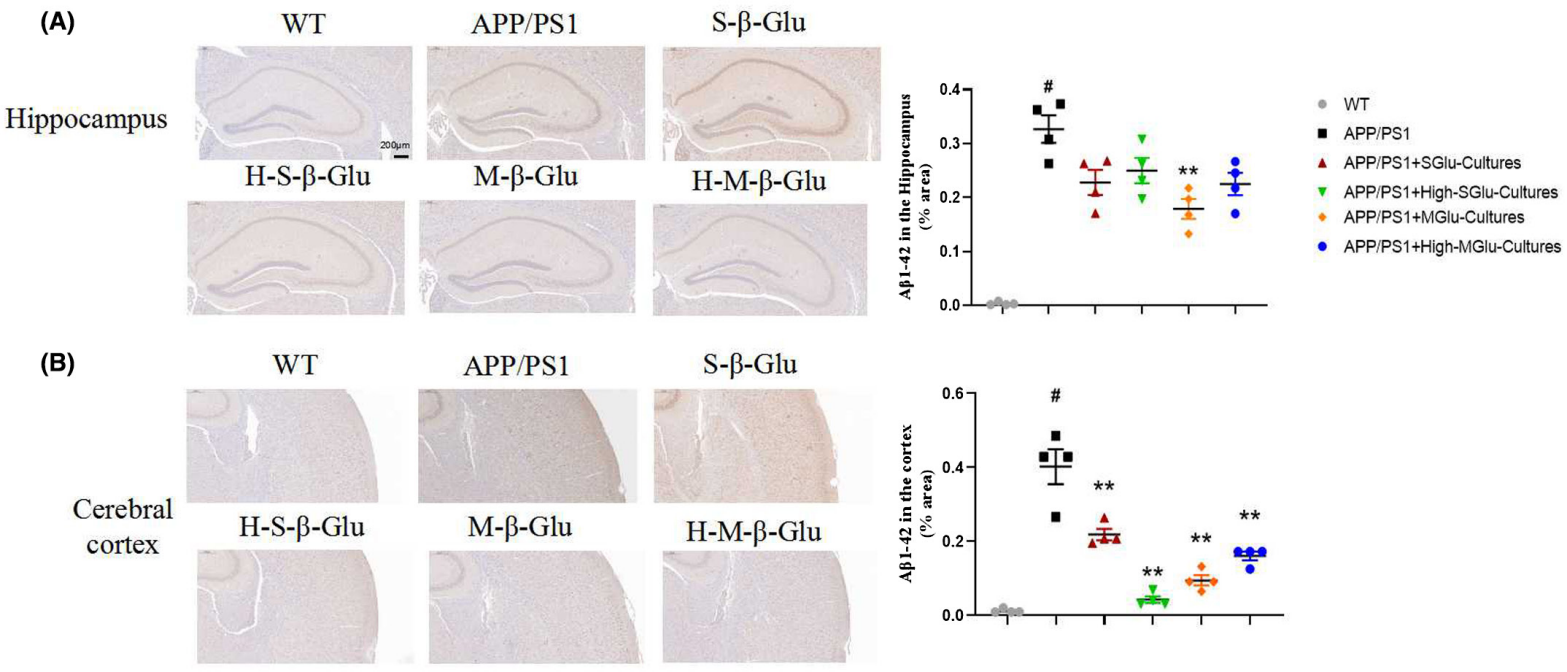
Gut microbiota cultures of β‐glucan‐treated mice alleviated AD‐related pathological changes in the hippocampus and cerebral cortex of APP/PS1 mice using H&E staining. (A,B) Imaging (left) and quantification (right) of Aβ1–42 by H&E staining in the hippocampus (A) and cerebral cortex (B). Scale bar represents 200 μm. Bars shown are means ± SEM. **p < 0.01 vs. APP/PS1; #*p* < 0.05 vs. WT; *n* = 4 mice/group.

**FIGURE 8:**
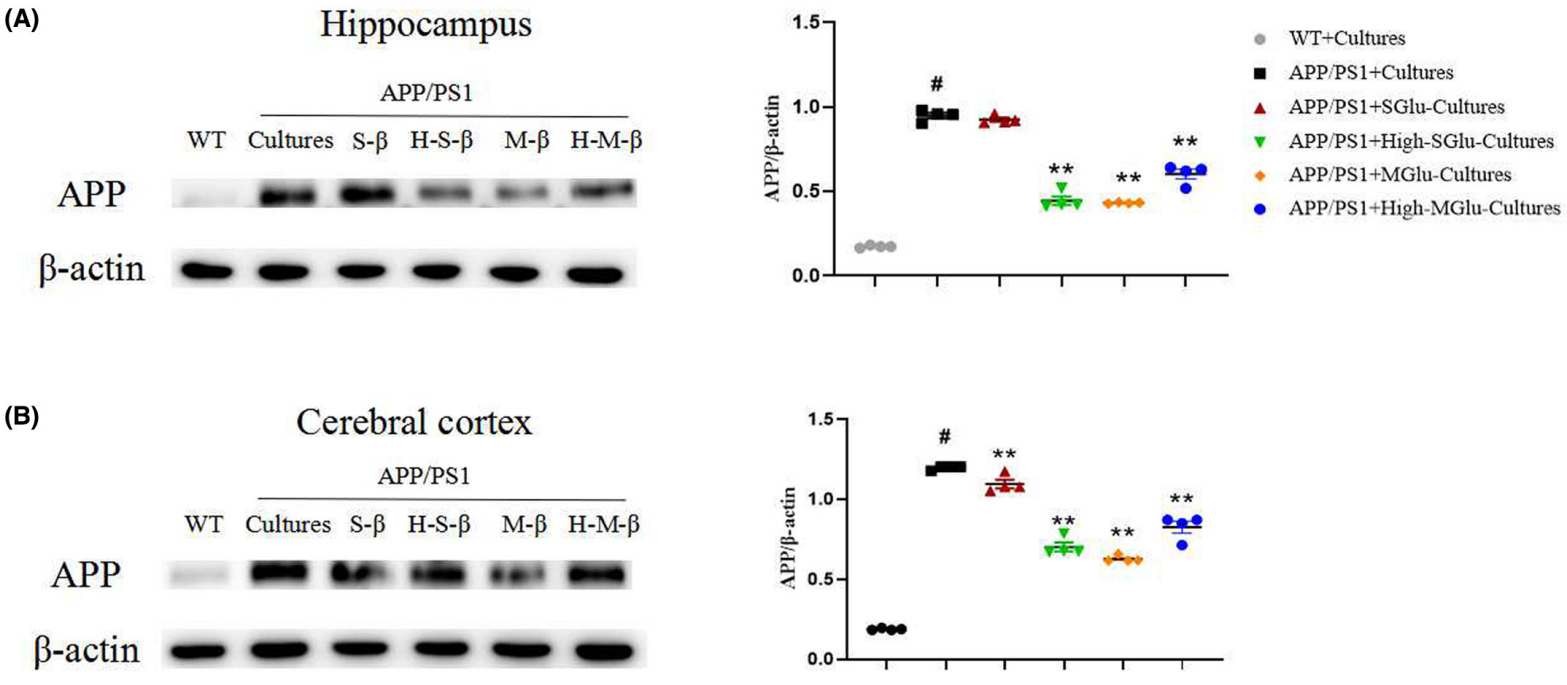
Gut microbiota cultures of β‐glucan‐treated mice decreased APP in the hippocampus and cerebral cortex of APP/PS1 mice using Western blotting. (A,B) Western blotting imaging (left) and quantification (right) of APP proteins in the hippocampus (A) and cerebral cortex (B). Data shown are means ± SEM. **p < 0.01 vs. APP/PS1; # *p* < 0.05 vs. WT; *n* = 4 mice/group.

**FIGURE 9:**
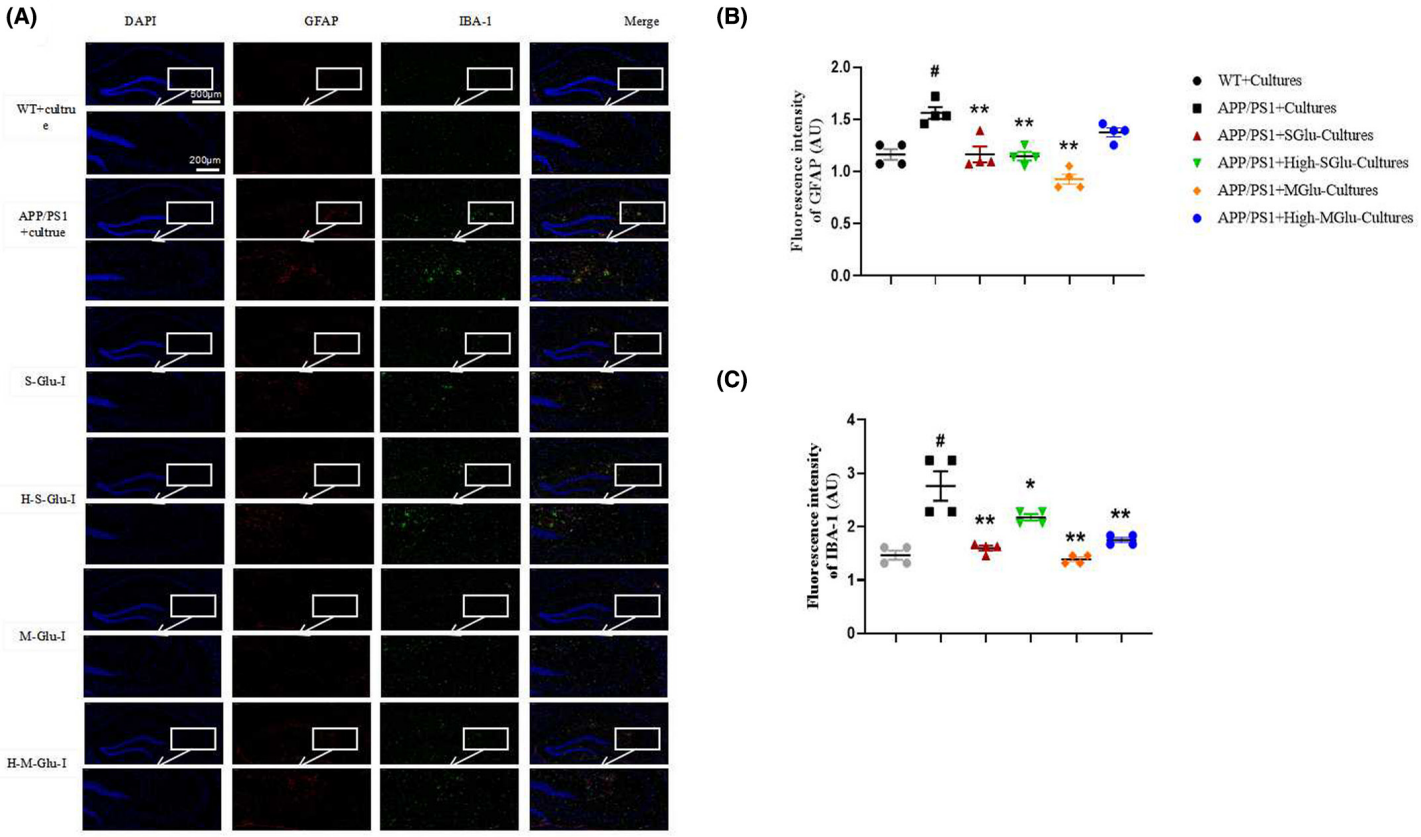
Gut microbiota cultures of β‐glucan‐treated mice reduced the activation of astrocytes and microglia in the brain of APP/PS1 mice using immunofluorescence. (A) Immunostaining of hippocampal slices with anti‐GAFP (red) and anti‐IBA1 (green). (B,C) The fluorescence intensity of GFAP, a marker of astrocyte activation (B) or IBA‐1, a marker of microglia activation (C). Scale bars represent 500 μm (upper) or 200 μm (lower in magnification). Bars shown are means ± SEM. *p < 0.05, ** *p* < 0.01 vs. APP/PS1; #*p* < 0.05 vs. WT; *n* = 4 mice/group.

**FIGURE 10:**
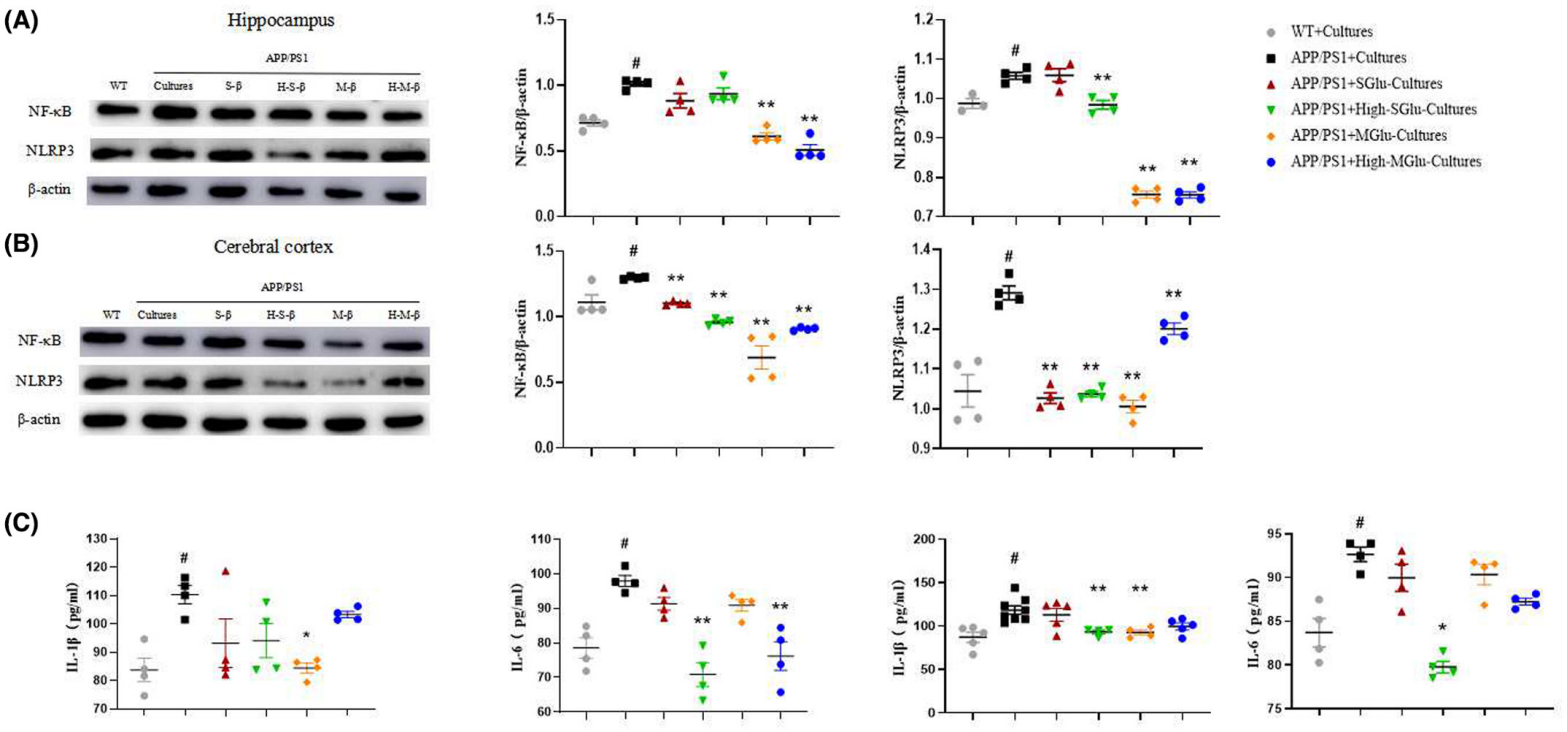
Gut microbiota cultures of β‐glucan‐treated mice attenuated inflammatory changes in the hippocampus and cerebral cortex of APP/PS1 mice using Western blotting and ELISA. (A–D) Western blotting imaging (A,C) and quantification (B,D) of NF‐κB (left) and inflammasome NLRP3 (right) in the hippocampus (A,B) and cerebral cortex (C,D). (E,F) The effect of intestinal flora cultures on the contents of IL‐1β (left) and IL‐6 (right) in the hippocampus (E) and cerebral cortex (F) by ELISA. Bars shown are means ± SEM. *p < 0.05, ** *p* < 0.01 vs. APP/PS1; # *p* < 0.05 vs. WT; *n* = 4 mice/group.

Immunohistochemical detection revealed that the intestinal cultures from APP/PS1 mice treated with β‐glucan significantly reduced Aβ plaque depositions in the hippocampus and cerebral cortex of APP/PS1 mice (Figure 7A,B). Consistent with this, the β‐glucan culture treatment also reduced the expression of APP in the hippocampus and cerebral cortex of APP/PS1 mice (Figure 8A,B).The intestinal cultures of APP/PS1 mice treated with β‐glucan not only significantly reduced the activation of microglia and astrocytes, as indicated by GFAP‐ and IBA‐1‐labeled cells, respectively, in the brain (Figure 9A–C), but also reduced the expression of NF‐kB and NLRP3 in the hippocampus and cerebral cortex, especially in the M‐Glu‐I and HM‐Glu‐I groups (Figure 10A–D). The intestinal cultures of APP/PS1 mice treated with β‐glucan tended to reduce the inflammatory factors IL‐1β and IL‐6, although the potency varied in different brain regions and assay indices (Figure 10E,F). Meanwhile, these measurements were not significantly changed after the animal's own gut microbiota cultures or vehicle administration to WT and APP/PS1 (Figure S2).

### 3.6 Effects of β‐glucan or intestinal flora cultures of β‐glucan‐treated mice on pathological changes in colon tissues of APP/PS1 mice

**FIGURE 11:**
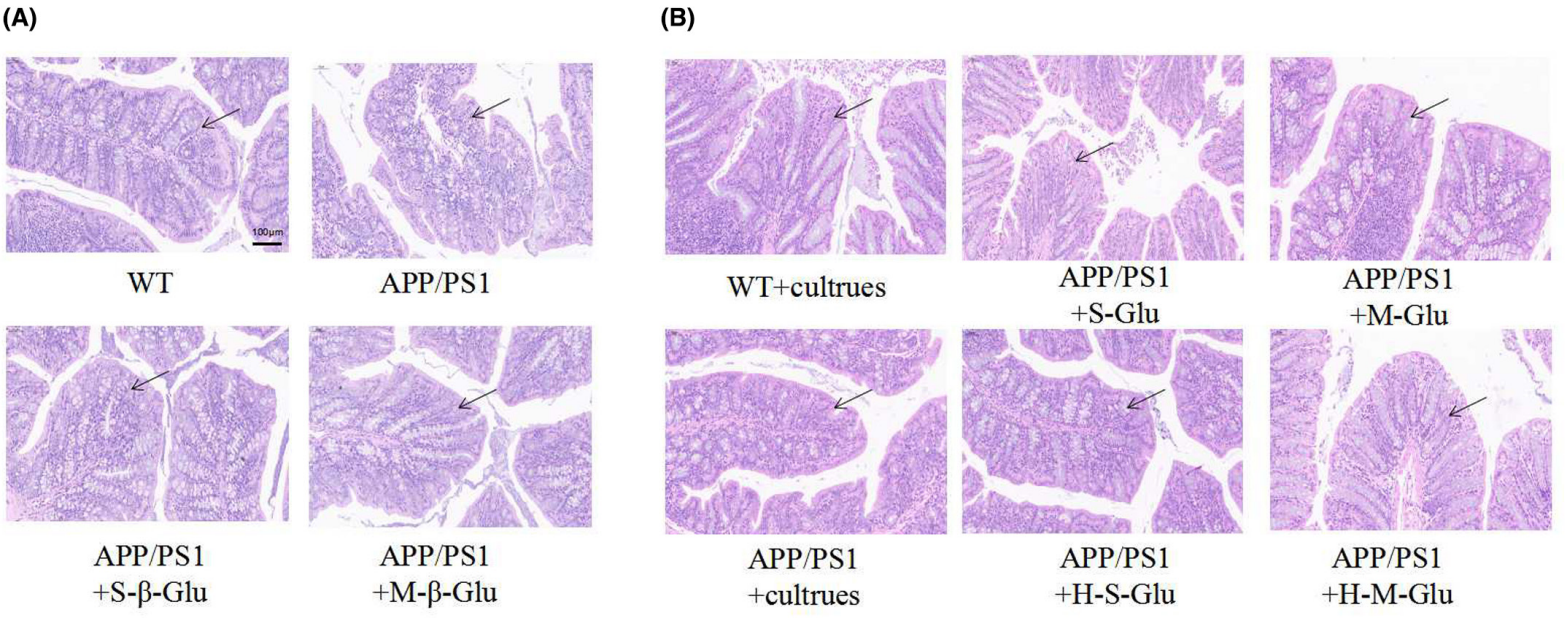
Treatment with β‐glucan (A) or gut microbiota cultures of β‐glucan‐treated mice (B) attenuated inflammatory responses by HE staining in APP/PS1 mice. (A) β‐Glucan restored the morphology of intestinal parietal cells in APP/PS1 mice. (B) Intestinal flora cultures of mice treated with β‐glucan also restored the morphology of intestinal parietal cells in APP/PS1 mice. Scale bar represents 100 μm.

Compared to the WT, the intestinal tissues of APP/PS1 mice were different, with the loose structure of intestinal tissues and some vacuoles in APP/PS1mice (Figure 11A); these were recovered after treatment with β‐glucan, in particular M‐β‐glucan. In contrast, the changes after culture treatment were not obvious between the groups (Figure 11B).

## 4 DISCUSSION

Animal studies have shown that yeast has neuroprotective effects on AD.^36^, ^37^, ^38^ More specifically, yeast increases the antioxidant capacity of supplementing selenium to resist AD.^39^ Further studies demonstrate that yeast β‐glucan, the main component of yeast cell wall, is related to the progression of AD.^40^ Yeast β‐glucan has a variety of biological effects, including anti‐inflammatory, immune regulation, and regulation of intestinal flora.^20^, ^41^ The function of β‐glucan is overall complicated. First, β‐glucan chelates copper ions to alleviate the symptom of AD.^42^ Second, β‐glucan is an immune agent that can improve the body's immunity.^43^, ^44^ Being recognized as an antigen, β‐glucan inhibits proliferation of harmful bacteria and changes the composition of the intestinal flora metabolites.^45^ Finally, β‐glucan can also be used as a substrate which is selectively taken by host microorganisms to change the metabolites of intestinal flora.^46^, ^47^ This can affect inflammation and cytokines through the brain‐gut axis which is beneficial to health.^48^, ^49^ Therefore, ingestion of β‐glucan can change the intestinal microecology and improve the pathophysiology of AD.

In the present study, we demonstrated that yeast β‐glucan affected the progression of AD in APP/PS1 mice. In behavioral tests, APP/PS1 mice showed obvious cognition impairment, which was significantly attenuated by treatment with β‐glucan. Consistent with this, β‐glucan reduced Aβ deposition and APP expression in the hippocampus and cerebral cortex of APP/PS1 mice. These are supported by the previous study in a mouse AD model induced by Aβ1‐42.^50^ It has been shown that β‐glucan from mushrooms improves the abnormality of learning and memory function and histopathology in the hippocampus in APP/PS1 mice via its enhancement of phagocytosis,^51^ leading to reduction of Aβ accumulation and toxicity.^52^

It is well known that β‐glucan does not cross the blood–brain barrier (BBB) and is difficult to be absorbed by the human body.^53^ Nevertheless, β‐glucan can improve the cognitive function in a mouse model of AD generated by Aβ injections,^40^ but the mechanism by which β‐glucan improves cognitive ability is not yet clear. The gut microbiota has been shown to affect the gut‐brain axis which in turn regulates cognition.^54^ Supplementing with probiotics, such as Bifidobacterium bifidum and Lactobacillus, significantly improves the cognitive ability of AD patients.^55^ In contrast, Aristobis and Spirulina, which are pathogens or opportunistic pathogens, are associated with inflammation in rodents.^56^, ^57^ Previous studies have found that the symbiotic microbiome of animals is a very important factor affecting host health^58^, ^59^ and intestinal flora transfer induced by long‐term broad‐spectrum antibiotic treatment mitigated brain Aβ deposition in APP/PS1 mouse models of AD.^60^ This suggests that the integrity of intestinal flora may be a key factor affecting the brain‐gut axis. In this study, the composition of intestinal flora in the four groups was significantly different, and the total number of intestinal flora and firmicutes in APP/PS1 mice were significantly reduced compared with those in WT, while β‐glucan reversed this situation to some extent.

The intestinal flora is closely related to SCFAs.^61^ For instance, Bifidobacteria in Firmicutes can produce glycosyl hydrolases which metabolize carbohydrates into SCFAs.^62^ SCFAs can change the intestinal environment and stimulate the brain‐gut axis to reduce inflammation. In addition, SCFAs also help maintain intestinal homeostasis, barrier, immunology, and host metabolism.^63^ Propionate can disrupt the assembly of Aβ peptides into neurotoxic oligomers^64^, protect the integrity of the BBB, and inhibit non‐specific inflammatory responses associated with microbial infections in vitro.^65^ In the present study, we demonstrated that β‐glucan could change microbial metabolites such as SCFAs by affecting the intestinal flora. In addition, compared with WT, APP/PS1 mice displayed significant decreases in the content of SCFAs in the colon, including acetic acid, propionic acid, valeric acid, and caproic acid. The contents of propionic acid, valeric acid, caproic acid, and butyric acid were increased after supplementing with S‐β‐glucan and the contents of the first three were increased following treatment with M‐β‐glucan. These results suggest that β‐glucan most likely regulates the metabolism of SCFAs through the intestinal flora.

In addition to SCFAs, bile acid, which is produced in the liver, is also an important component of the contents of the colon. Bile acid is metabolized by enzymes produced by intestinal bacteria and is important for maintaining healthy intestinal microbiota, balancing lipid, sugar metabolism, and insulin sensitivity. Bile acids is divided into two categories: primary and secondary bile acids according to their sources. The secondary bile acid is formed by the primary bile acid through the action of intestinal bacteria. Therefore, intestinal microbes can regulate the production of secondary bile acids, thereby affecting signal transmission.^66^ In the present study, we found that after the total amount of bacteria in the intestinal flora of APP/PS1 mice was reduced, its primary bile acids such as cholic acid were significantly higher than those of WT mice, indicating that the ability of APP/PS1 mice to decompose bile acids was decreased. After supplementation with β‐glucan, the primary bile acid content of APP/PS1 mice was decreased, suggesting that β‐glucan improves the ability to decompose bile acids after improving the intestinal microecology.

However, it is still unclear how yeast β‐glucan can improve cognitive ability by regulating the intestinal microecology. We observed through correlation analysis that its underlying mechanism may be related to inflammatory signal pathways. Recently, more and more functional studies have shown that neuroinflammation accelerates the progress of cell death and AD.^67^ It has been demonstrated that SCFAs reduce the activation of NF‐kB,^68^ indicating anti‐inflammatory activity. This was supported by the changes in NLRP3 inflammasomes, which were highly expressed in APP/PS1 mice. NLRP3 knockout or dysfunction of its key signal components significantly reduces Aβ‐induced microglia activation in vitro^69^ and reverses cognitive impairment in AD mouse models.^70^ Our data showed that APP/PS1 mice had obvious inflammatory responses, which were significantly reduced by supplementation with β‐glucan; more specifically, β‐glucan produced significant decreases in the expression of P65 and NLRP3 in the hippocampus and cerebral cortex, activation of microglia and astrocytes, and cytokines such as IL‐1β and IL‐6 in brain tissues. Of note, IL‐1β, an inflammatory cytokine, can inhibit the phagocytosis of Aβ by microglia (Ising et al., 2019;.^71^ It was noted that the cytokines in the serum did not change markedly, indicating that β‐glucan attenuates brain inflammation primarily by SCFAs through the brain‐gut axis.

This study demonstrated that yeast β‐glucan significantly improved cognitive function in AD mice. We hypothesized that β‐glucan changed the intestinal microbiome and intestinal flora metabolites to reduce brain inflammation. In order to test this hypothesis, we examined the intestinal cultures of mice treated with β‐glucan transplantation via rectal administration. It has been shown that transplantation of feces from healthy mice to mice with spinal cord injury promotes regeneration of neuronal axons and enhances the integrity of the intestinal barrier and gastrointestinal motility.^72^ Further studies have shown that APP/PS1 mice display beneficial changes in the intestinal flora and SCFAs following fecal transplantation and, more importantly, delay the onset of cognition deficits.^73^ The research of fecal bacteria transplantation has achieved success in clinical practice, and fecal bacteria transplantation has played an effective role in autism.^74^ Since fecal transplantation mainly assesses the impact of intestinal flora, we further examined the impact of intestinal flora metabolites by using in vitro cultures of the colon and intestines of each group of mice in the second part of the experiment. The cultures were administered rectally to both WT and APP/PS1 mice. It was interesting to note that WT and APP/PS1 mice did not show any significant changes after receiving their own intestinal content cultures or equal‐dose vehicles (anaerobic meat liver soup medium), suggesting that the effect of supplementing metabolites of autologous intestinal flora on the progression of AD is excluded. In contrast, APP/PS1 mice supplemented rectally with the cultures of from β‐glucan‐treated mice, especially those from HS‐β‐Glu and M‐β‐Glu, displayed significant enhancement of the memory function and decreases in inflammatory responses and activation of microglia and astrocytes. These suggest that the metabolites of the intestinal flora such as SCFAs may play an important role in the development of AD.

The intestinal barrier between the internal environment of the intestine and the materials in the cavity is formed by the intestinal epithelium. Its function is to effectively absorb nutrients and prevent the absorption of microorganisms, harmful antigens and toxins.^75^, ^76^ The integrity of intestinal cells is likely related to the progression of AD. Once the intestinal barrier is broken, Gram‐negative bacteria can cause brain Aβ deposits.^60^, ^77^ We showed that APP/PS1 mice had loose intestinal tissue structure and occasional vacuoles, which were obviously different from WT controls. Supplementation of β‐glucan was beneficial to the proliferation of intestinal wall cells and the restoration of intestinal microecology; this was supported by previous studies.^78^ However, the effect of supplementation of colonic metabolites on intestinal cells was not significant, indicating that the proliferation of intestinal wall cells may be stimulated by the effect of β‐glucan.

## 5 CONCLUSIONS

The present study provided solid results supporting that yeast β‐glucan improves cognition, attenuates neuroinflammation, restores the steady‐state composition of the microbial community, changes the composition of metabolites of the intestinal flora, and maintains the integrity of the intestinal barrier associated with AD. These may be related to the intestinal flora metabolites such as SCFAs and bile acids. Therefore, yeast β‐glucan which improves the intestinal flora metabolites can be a promising strategy to treat or even prevent AD.

## FUNDING INFORMATION

This work was supported by Academic Promotion Program of Shandong First Medical University (Grand No. 2019QL011); National Natural Science Foundation of China (Grand No. 81773717, 82073827); Shandong Provincial Natural Science foundation (Grand No. ZR2020MH412);TCM Science and Technology Development Plan of Shandong Province(Grand No. 2017–241).

## CONFLICT OF INTEREST STATEMENT

The authors declared that they have no conflicts of interest to this work. We declare that we do not have any commercial or associative interest that represents a conflict of interest in connection with the work submitted.

## Supporting information


Figure S1.

Figure S2.

## Data Availability

All data generated or analyzed in this study are included in this article.
