# Supplementary figures and images for: β‐Glucan attenuates cognitive impairment of APP/PS1 mice via regulating intestinal flora and its metabolites
Source: CNS Neurosci Ther. 2023 Mar 8;29(6):1690–704. doi: 10.1111/cns.14132 (PMC10173722; doi:10.1111/cns.14132)

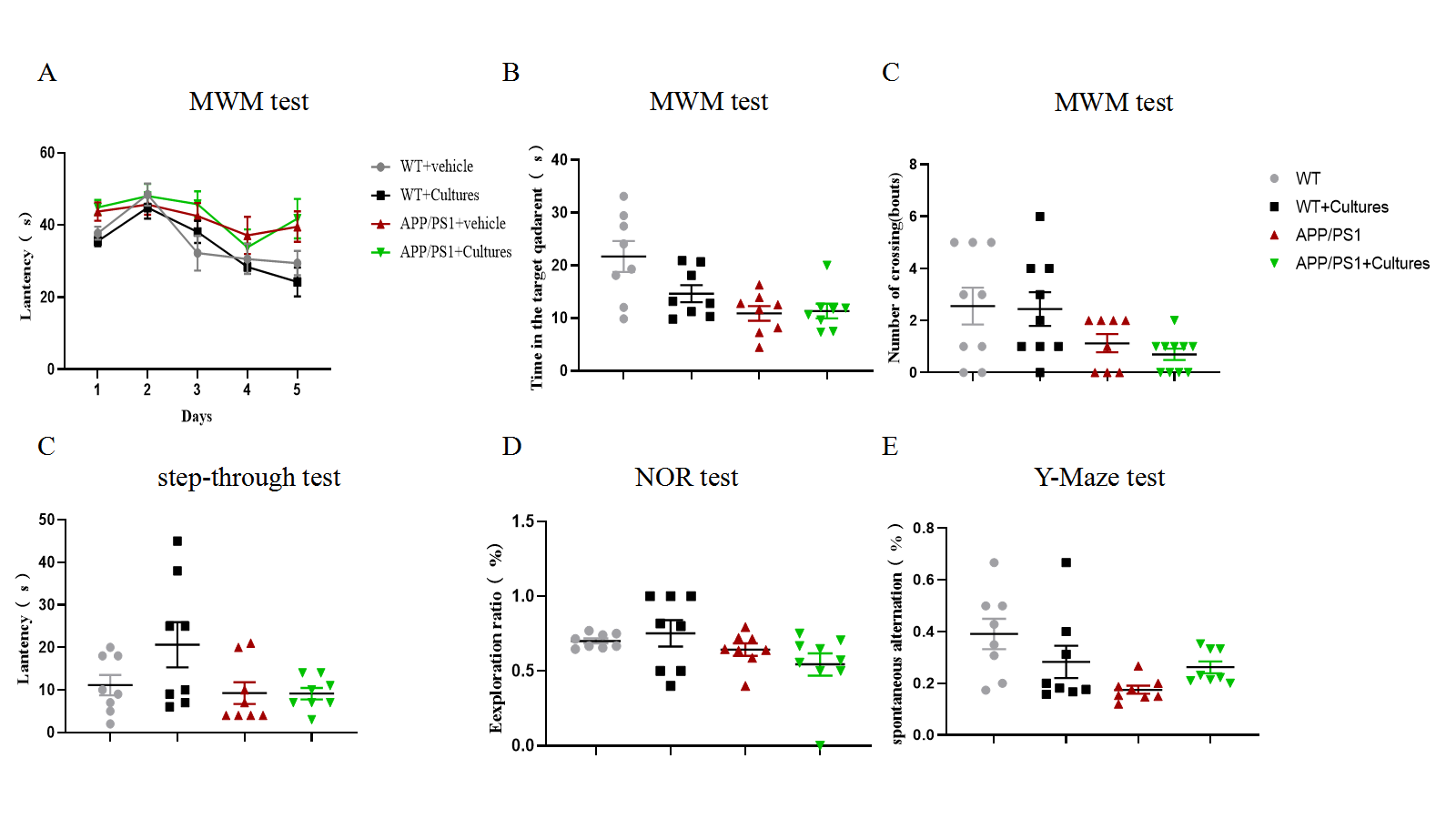

Supplement: Supplementary file 1 — Figure S1. [file CNS-29-1690-s001.tif]

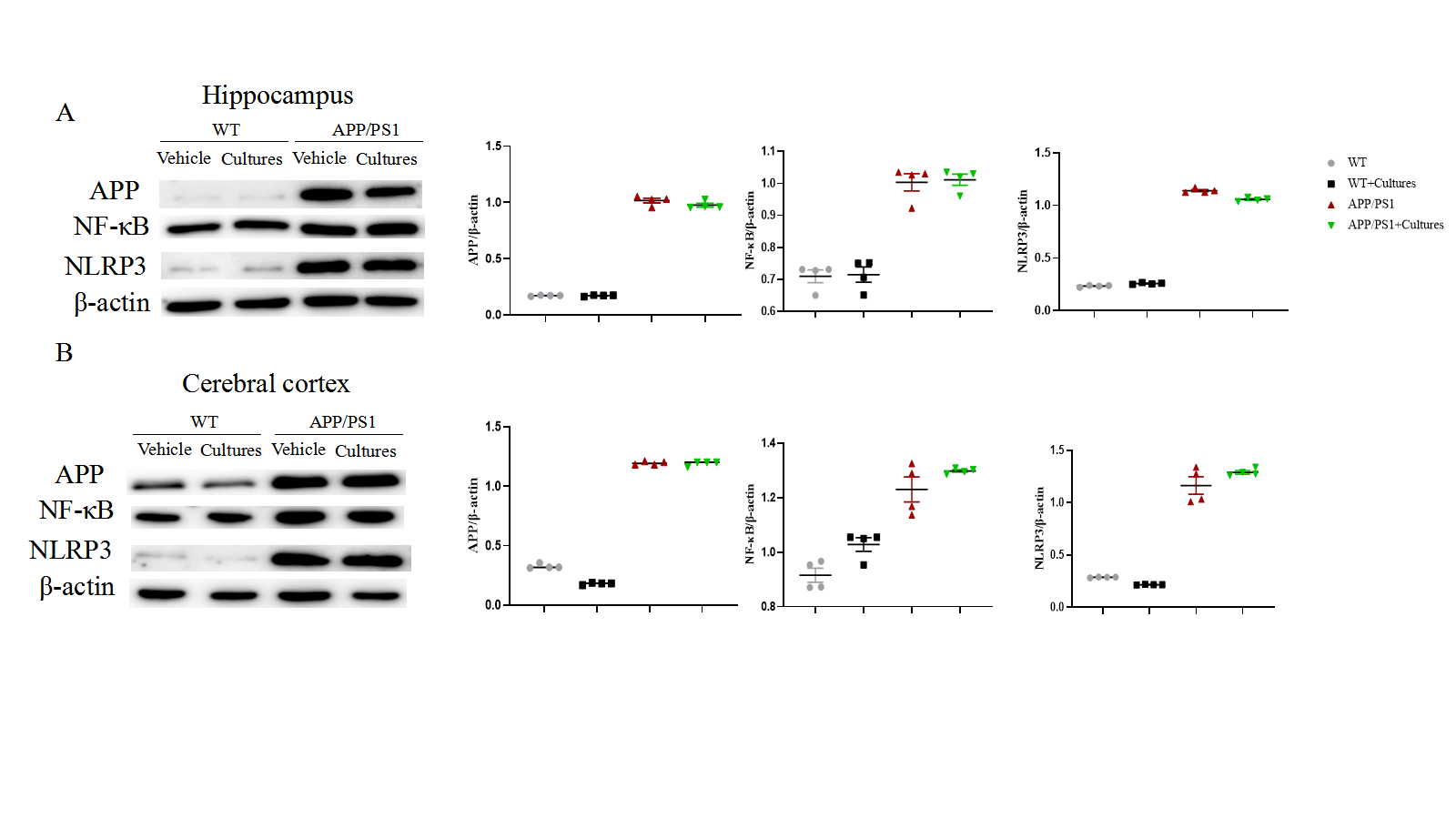

Supplement: Supplementary file 2 — Figure S2. [file CNS-29-1690-s002.tif]
